# Role of a Genetic Polymorphism in the Corticotropin-Releasing Factor Receptor 1 Gene in Alcohol Drinking and Seeking Behaviors of Marchigian Sardinian Alcohol-Preferring Rats

**DOI:** 10.3389/fpsyt.2013.00023

**Published:** 2013-04-12

**Authors:** Lydia O. Ayanwuyi, Francisca Carvajal, Jose M. Lerma-Cabrera, Esi Domi, Karl Björk, Massimo Ubaldi, Markus Heilig, Marisa Roberto, Roberto Ciccocioppo, Andrea Cippitelli

**Affiliations:** ^1^Pharmacology Unit, School of Pharmacy, University of CamerinoCamerino, Italy; ^2^Laboratory of Clinical and Translational Studies, National Institutes of Health, National Institute on Alcohol Abuse and AlcoholismBethesda, MD, USA; ^3^Committee on the Neurobiology of Addictive Disorders, The Scripps Research InstituteLa Jolla, CA, USA

**Keywords:** CRF, SNP, self-administration, msP, yohimbine, relapse

## Abstract

Marchigian Sardinian alcohol-preferring (msP) rats exhibit innate preference for alcohol, are highly sensitive to stress and stress-induced alcohol seeking. Genetic analysis showed that over-expression of the corticotropin-releasing factor (CRF) system of msP rats is correlated with the presence of two single nucleotide polymorphisms (SNPs) occurring in the promoter region (position −1836 and −2097) of the CRF1 receptor (CRF1-R) gene. Here we examined whether these point mutations were associated to the innate alcohol preference, stress-induced drinking, and seeking. We have recently re-derived the msP rats to obtain two distinct lines carrying the wild type (GG) and the point mutations (AA), respectively. The phenotypic characteristics of these two lines were compared with those of unselected Wistar rats. Both AA and GG rats showed similar patterns of voluntary alcohol intake and preference. Similarly, the pharmacological stressor yohimbine (0.0, 0.625, 1.25, and 2.5 mg/kg) elicited increased operant alcohol self-administration under fixed and progressive ratio reinforcement schedules in all three lines. Following extinction, yohimbine (0.0, 0.625, 1.25, and 2.5 mg/kg) significantly reinstated alcohol seeking in the three groups. However, at the highest dose this effect was no longer evident in AA rats. Treatment with the CRF1-R antagonist antalarmin (0, 5, 10, and 20 mg/kg) significantly reduced alcohol-reinforced lever pressing in the AA line (10 and 20 mg/kg) while a weaker or no effect was observed in the Wistar and the GG group, respectively. Finally, antalarmin significantly reduced yohimbine-induced increase in alcohol drinking in all three groups. In conclusion, these specific SNPs in the CRF1-R gene do not seem to play a primary role in the expression of the msP excessive drinking phenotype or stress-induced drinking but may be associated with a decreased threshold for stress-induced alcohol seeking and an increased sensitivity to the effects of pharmacological blockade of CRF1-R on alcohol drinking.

## Introduction

Alcoholism is an etiologically and clinically heterogeneous disorder in which compulsive alcohol use and elevated vulnerability to relapse represent core symptoms (McLellan et al., 1992). Exposure to alcohol is a necessary precondition for development of alcoholism. However, environment and heritability factors play a dramatic role in controlling individual predisposition to developing alcohol abuse (Cloninger et al., 1981; Schuckit et al., 1985; Enoch and Goldman, 1999; Lovinger and Crabbe, 2005). Environmental stress has been recognized as one of the major factors for alcohol abuse and dependence (Pohorecky, 1991; Sarnyai et al., 2001; Sinha, 2001; Shaham et al., 2003; Breese et al., 2005b). However, the interaction between environmental stress and heritable factors in the development of alcoholism is still largely unexplored. Understanding the nature of this interaction in regulating individual risk of becoming an alcohol abuser represents a major challenge in this research field and may provide invaluable help for the development of preventive strategies or pharmacotherapeutic remedies.

Studies conducted in our laboratory demonstrated that genetically selected Marchigian Sardinian alcohol-preferring (msP) rats show excessive daily alcohol drinking (6–8 g/kg body weight) in a binge-type pattern, leading to blood alcohol levels as high as 100–120 mg/dl (Ciccocioppo et al., 2006). This selected rat line is highly sensitive to stress and stress-induced alcohol seeking (Ciccocioppo et al., 2006), demonstrates an anxious phenotype (Hansson et al., 2006), and has depressive-like symptoms that recover following alcohol consumption (Ciccocioppo et al., 1999). Hence, these animals may represent a preclinical model of genetic predisposition to high alcohol drinking and relapse endowed with significant predictive validity. In addition, msP rats appear to share important common characteristics with the human disease that also confer to them important elements of face and construct validity (Ciccocioppo et al., 2006; Ciccocioppo, 2013).

The corticotropin-releasing factor (CRF) is a 41 amino acid peptide that integrates many of the endocrine, behavioral, and autonomic responses to stress (Sarnyai et al., 2001). CRF has been implicated in alcohol addiction because there is evidence that neuroadaptive changes triggered by a prolonged history of alcohol exposure lead to a chronically dysregulated CRF/CRF1 receptor (CRF1-R) system activity that, in turn may drive excessive and uncontrolled alcohol consumption motivated by relief of negative emotionality (Heilig and Koob, 2007; Koob, 2010; Breese et al., 2011). In particular, upregulation of the peptide has been observed in the extended amygdala during alcohol withdrawal (Merlo Pich et al., 1995; Zorrilla et al., 2001; Olive et al., 2002; Roberto et al., 2010) and long-term upregulation of CRF1-Rs has been also shown in these structures in animals with a previous history of alcohol dependence (Sommer et al., 2008). Similarly, msP animals show innate upregulation of CRF1-R expression and density in multiple corticolimbic regions, indicating hyperfunction of the CRF system (Hansson et al., 2006), which is attenuated by alcohol consumption (Hansson et al., 2007). In agreement with these findings, both alcohol-induced neuroadaptations leading to dysregulated CRF system and the innate hyperfunction of the system in msP rats have been shown to confer sensitivity to the treatment with CRF1-R antagonists. Core symptoms of alcohol dependence including excessive alcohol self-administration and stress-induced relapse to alcohol seeking were in fact attenuated at doses that had no effects in non-dependent unselected animals (Funk et al., 2006a; Hansson et al., 2006; Sabino et al., 2006; Gehlert et al., 2007; Ciccocioppo et al., 2009). All these similarities suggest that innate upregulation of CRF1-R expression mimics the post-dependent phenotype such that msP rats have been proposed as phenocopies of post-dependent animals (Heilig and Koob, 2007).

Further work done in msP rats provided evidence that excessive alcohol drinking and stress vulnerability may be associated with the occurrence of two single nucleotide polymorphisms (SNPs) in the promoter region (position −1836 and −2097) of the gene encoding the CRF1 receptor, an observation that closely correlated with innate upregulation of the CRF1-R transcript (Hansson et al., 2006). Genetic variation at the CRF1-R locus as a susceptibility factor for excessive alcohol drinking might have parallels in humans, where a similar association was reported (Treutlein et al., 2006). It is, however, unclear whether the −1836 and −2097 SNPs are causally related to escalated alcohol consumption. Of note, high alcohol preference is a complex trait, and may be driven by different genetic factors in different genetically selected preferring lines. These SNPs are unique to msP animals, and genetic screening in the Indiana alcohol-preferring [P (Li et al., 1991)] and the Sardinian alcohol-preferring [sP (Colombo et al., 1995)] indicates that these lines do not carry mutations at the CRF1-R locus (oral communication).

Here, we tested whether the occurrence of the SNPs is responsible for the high alcohol drinking and preference of msP rats and whether the occurrence of the point mutations may contribute to other behavioral differences including sensitivity to the treatment with CRF1-R antagonist and relapse susceptibility. To assess how environmental stress interacts with heritable factors, rats were re-derived from the original msP line to obtain two distinct lines, one carrying the two point mutations (AA) and the wild type line (GG). The phenotypic characteristics of these two msP rat lines were assessed following stress exposure and compared with those of unselected Wistar rats.

## Materials and Methods

### Animals

Subjects were adult males from two distinct sub-lines derived from the original msP line (65th generation). Animals were bred at the animal facility of the University of Camerino, Italy. Breeding started following genetic screening of the promoter region encoding for CRF1-Rs. Sequence variation AA versus GG in position −1836 and −2097 respectively, of the CRF1-R transcript distinguished the two msP lines. Specifically, 80 msP rats were sequenced using Taqman-PCR analysis of tail DNA to identify animals carrying (AA) or not carrying (GG) both variants. The homozygous male and female AA and GG were then bred to obtain re-derived lines selectively carrying the AA and the GG types. They were bred for two more generations and then animals from the third and fourth generations were used for experiments. Male Wistar rats (Charles River, Calco, Italy) were employed as a behavioral control. All rats (350–450 g) at the time of the experiments were housed in groups of five or four except where otherwise specified, on a reverse 12 h light-dark cycle (lights off at 08:30 AM) at a constant temperature of 20 ± 2°C and relative humidity of 45–55%, with free access to tap water and food pellets (4RF18, Mucedola, Settimo Milanese, Italy). Animals were handled three times before the onset of each experiment. All procedures followed the *EU Directive for Care and Use of Laboratory Animals*.

### Drugs

Alcohol solution (10% v/v) was prepared by diluting 95% alcohol (F.L. Carsetti s.n.c.-Camerino) in tap water. The selective CRF1-R antagonist antalarmin (*N*-butyl-*N*-ethyl-[2,5,6-trimethyl-7-(2,4,6-trimethylphenyl)-7*H*-pyrrolo[2,3-d]pyrimidin4-yl]-amine (Webster et al., 1996) was obtained from the National Institute on Alcohol Abuse and Alcoholism (NIAAA/NIH). Antalarmin was suspended in a vehicle composed of 10% Tween 80 and distilled water and given intraperitoneally (i.p.) in a 1 ml/kg volume injection. Yohimbine hydrochloride (17-hydroxyyohimban-16-carboxylic acid methyl ester hydrochloride) was purchased from Sigma (Sigma-Aldrich, Italy) and dissolved in distilled water. Yohimbine was administered i.p. in a 1 ml/kg volume injection. Physiological saline was injected three times prior to drug testing for habituation to the experimental procedures.

### Two-bottle free choice drinking paradigm

To ascertain the relation of CRF1-R promoter genotype to home cage alcohol intake, AA (*n* = 8) and GG (*n* = 8) msP rats were used and their intake measured daily. Rats were single-housed to provide accurate record of home cage drinking. Animals were provided *ad libitum* concurrent, continuous access to 10% alcohol solution, water, and food pellets. Fluids were presented in graduated plastic bottles equipped with a stainless-steel drinking spouts inserted through two grommets in front of the cage and were changed daily at 90–120 min into the dark period of the light/dark cycle. The placement of the alcohol bottle was alternated daily to control for side preference. This procedure was carried out for 15 days. Data are presented as daily alcohol intake (g/kg) and percentage of alcohol preference [100× alcohol intake (ml)/total fluid intake (ml)].

### Operant self-administration apparatus and training

Training and testing were conducted in operant conditioning chambers housed in sound-attenuating cubicles (Med Associates Inc., Georgia, VT, USA). Each operant chamber was equipped with two retractable levers positioned laterally to a drinking reservoir. Visual stimuli were presented via a light located on the back panel. A microcomputer controlled the delivery of the fluids, presentation of visual stimuli, and recording of the behavioral data. Rats were trained to self-administer 10% alcohol (v/v) in 30 min daily sessions on a fixed ratio 1 (FR-1) schedule of reinforcement, in which each response on the active lever resulted in delivery of 0.1 ml of fluid. A response on the second lever had no programed consequences. For the first 3 days, rats were allowed to lever-press for a 0.2% (w/v) saccharin solution, and were then trained to self-administer 10% alcohol by gradually increasing the percentage of alcohol and fading out the saccharin (Cippitelli et al., 2008).

### Operant alcohol self-administration on a fixed ratio 3 schedule of reinforcement following stress exposure

Rats (*n* = 34; 10 Wistars, 14 GG, and 10 AA msPs) were trained to self-administer 10% alcohol as described above. When all the rats reached the 10% alcohol stage, the schedule of reinforcement was changed from FR-1 to FR-3. Here, following three responses that delivered a reinforcer, a 5-s time-out period was in effect, during which responses were recorded but not reinforced. Once stable self-administration responding was obtained under this reinforcement schedule, the experiment was started. Stress exposure consisted of the challenge with the pharmacological stressor yohimbine at doses previously shown to increase alcohol-reinforced lever pressing in unselected Wistar animals (Marinelli et al., 2007). Yohimbine (0.0, 0.625, 1.25, and 2.5 mg/kg) was administered 30 min prior to the 30 min self-administration session. The experiment was conducted in parallel for the three rat lines using a Latin square counterbalanced within-subjects design. Test sessions were 4 days apart. Following each test session day, animals were allowed 1 day off, and a new baseline was then established over the following 2 days as previously reported (Cippitelli et al., 2010b). Results are described as number of rewards in 30 min.

### Operant alcohol self-administration on a progressive ratio schedule of reinforcement following stress exposure

Additional rats (*n* = 30; 10 Wistars, 10 GG and 10 AA msPs) were trained to self-administer 10% alcohol. When all the rats reached the 10% alcohol stage, the schedule of reinforcement was changed from FR-1 to FR-3. As described above, following three responses that delivered a reinforcer, a 5-s time-out period was in effect, during which responses were recorded but not reinforced. Once stable self-administration responding was obtained under this reinforcement schedule, the three rat lines were tested under a progressive ratio (PR) schedule of reinforcement to measure the break point, defined as the last ratio completed by the animal (Cippitelli et al., 2007; Karlsson et al., 2012), to obtain 10% alcohol following stress exposure. For this purpose, the response requirement (i.e., the number of lever responses or the ratio required to receive one dose of 10% alcohol) was increased as follows: for each of the first four alcohol deliveries the ratio was increased by 1; for the next four deliveries the ratio was increased by 2 and for all of the following deliveries the ratio was increased by 4. Each alcohol-reinforced response resulted in the house light being turned on for 1 s, whereas sessions were terminated when more than 30 min had elapsed since the last reinforced response. The experiment was conducted in parallel for the three rat lines using a Latin square counterbalanced within-subjects design. The pharmacological stressor yohimbine at the dose of 0.625 mg/kg or its vehicle were administered 30 min prior to PR testing. Test sessions were 4 days apart. Following each test session day, animals were allowed 1 day off, and a new baseline was then established over the following 2 days.

### Operant alcohol self-administration on FR-3 schedule: Effect of antalarmin

Other rats (*n* = 33; 7 Wistars, 12 GG and 14 AA msPs) were trained to self-administer 10% alcohol as described above. Schedule of reinforcement was switched from FR-1 to FR-3. Following three responses that delivered a reinforcer, a 5-s time-out period was in effect, during which responses were recorded but not reinforced. Once stable self-administration was obtained under the FR-3 reinforcement schedule, treatment with the CRF1-R antagonist antalarmin was started. The experiment was conducted by using a Latin square counterbalanced design. Antalarmin at doses of 5, 10, and 20 mg/kg or its vehicle were administered 30 min prior to sessions. Test sessions were 4 days apart. Following each test session day, animals were allowed 1 day off, and a new baseline was then established over the following 2 days. Results are described as number of rewards in 30 min.

### Effect of antalarmin on yohimbine-induced increase of alcohol-reinforced lever pressing (FR-3)

A new cohort of rats (*n* = 33; 8 Wistars, 10 GG and 15 AA msPs) was trained to self-administer 10% alcohol as described above. When stable baseline of responding was obtained under the FR-3 reinforcement schedule that included the 5 s time-out period, drug treatment started. In this experiment, we pre-treated the three rat lines either with the selective CRF1-R antagonist antalarmin or its vehicle prior to the injection of yohimbine (0.625 mg/kg) or yohimbine vehicle. Pre-treatments were given 30 min prior to treatments that in turn occurred 30 min prior to testing sessions. These testing sessions were conducted every fourth day using a Latin square counterbalanced design and occurred 4 days apart in which animals were allowed 1 day off, and a new baseline was then established over the following 2 days. Results are described as number of rewards in 30 min.

### Reinstatement induced by stress exposure

A new cohort of animals (*n* = 24; 7 Wistar rats, 8 GG and 9 AA msP rats) was trained at the same time to self-administer alcohol as described above. When 10% alcohol became available, the FR-1 schedule slightly changed such that each lever pressing was accompanied by the illumination of the house light for 5 s. During this time-out period response were recorded but not reinforced. 10% alcohol sessions lasted 30 min and were conducted for 15 days. Then, rats were subjected to 30 min daily extinction sessions for additional 15 consecutive days. During extinction the lever presses were no longer associated with alcohol delivery, but house light was still presented to allow for its concomitant extinction. Stress exposure consisted of the challenge with the pharmacological stressor yohimbine at doses previously shown to produce reinstatement to alcohol seeking in unselected Wistar rats (Le et al., 2005; Marinelli et al., 2007; Cippitelli et al., 2010a). Yohimbine (0.0, 0.625, 1.25, and 2.5 mg/kg) was administered 30 min prior to the 30 min reinstatement session that was conducted under identical condition of extinction sessions. A Latin square counterbalanced design was used. Test sessions were 4 days apart and conducted after three consecutive extinction sessions. Results are described as total number of responses in 30 min.

### Statistical analysis

All drug testing experiments were here analyzed by means of a two-way analysis of variance (ANOVA) with “drug treatment” as the within-subject factor and “rat line” as the between-subject factor. When appropriate, analyses were followed up by Fisher’s least significant difference (LSD) *post hoc* tests. The same statistical approach was employed to analyze drinking patterns of intake and preference of GG versus AA msP rat lines with the exception that “rat line” was the between-subject factor and “day” was used as the within-subject factor.

## Results

### Minimal changes in voluntary alcohol intake and preference of GG and AA msP rats

**Figure 1:**
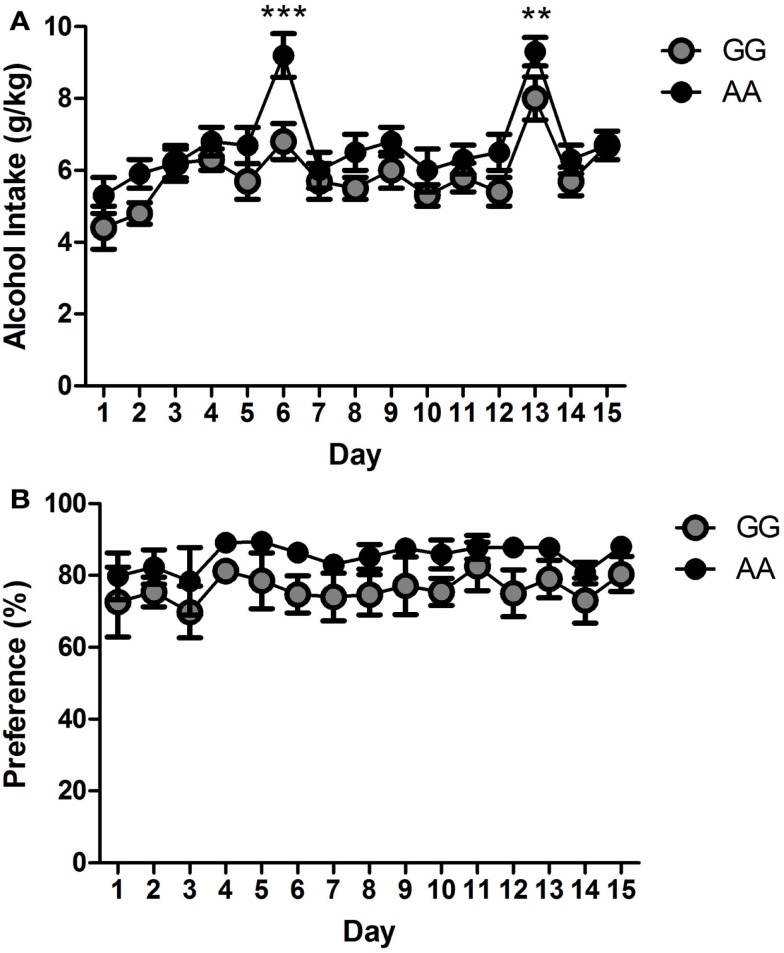
**Elevated alcohol drinking of the two msP lines GG (*n* = 8) and AA (*n* = 8) derived from the original msP line as assessed in the two-bottle free choice drinking paradigm**. GG and AA msP rats show minimal changes in **(A)** drinking patterns and **(B)** alcohol preference across a period of 15 days. Values are presented as the daily mean g/kg of alcohol intake (±SEM) and percent (%) of alcohol preference (±SEM), respectively. **p < 0.01 and ***p < 0.001, significant difference between the two msP rat lines. GG: gray line; AA: black line. For detailed statistics, see “Results.”

The GG and AA msP animals show a similar pattern of alcohol intake and preference over a period of 15 days as shown in Figure 1. Overall ANOVA failed to revealed a main effect of “line” [*F*(1,14) = 2.4, NS]. However, there was a main effect of “day” [*F*(14,196) = 24.4, *p* < 0.001], accompanied by interaction “line × day” [*F*(14,196) = 2.2, *p* < 0.01] to suggest minimal changes in voluntary alcohol intake across the 15-day exposure. Indeed, *post hoc* analysis showed difference in alcohol drinking between the two msP lines only on day 6 and 13 (*p* < 0.001 and *p* < 0.01, respectively, Figure 1A).

Data analysis of alcohol preference only showed difference in the main effect of “day” [*F*(14,196) = 24.4, *p* < 0.01] while failing to reveal significant difference in the main effect of “line” [*F*(1,14) = 2.4, NS] and interaction “line × day” [*F*(14,196) = 0.2, NS]. However, a slight and non-significant trend to a higher alcohol preference of the AA line compared to the GG line was observed (Figure 1B).

In a separate experiment, a different batch of the two msP lines was subjected to a two-bottle free choice drinking across a 50 day exposure. Results generally paralleled those shown here, that is no major difference between lines on patterns of 10% voluntary alcohol drinking and preference was found.

### Yohimbine similarly increases operant alcohol self-administration under a fixed ratio schedule of reinforcement in Wistar, as well as GG, and AA msP rats

**Figure 2:**
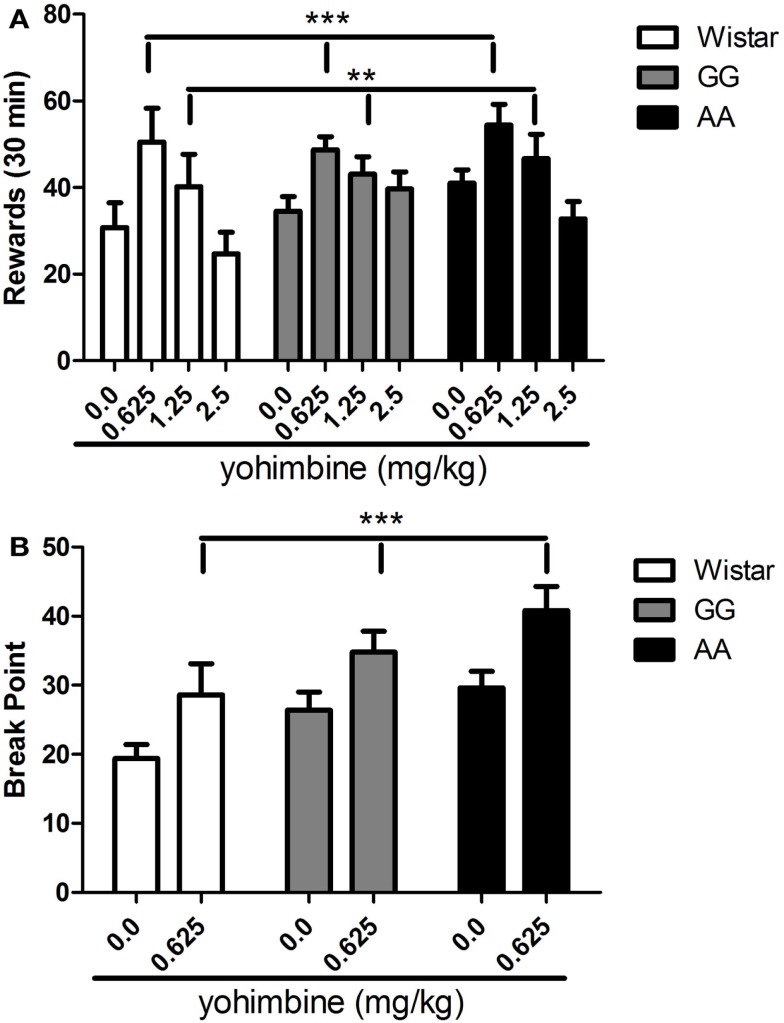
**(A)** Operant alcohol self-administration in Wistar (*n* = 10), as well as GG (*n* = 14), and AA (*n* = 10) msP rats under a fixed ratio 3 (FR-3) schedule of reinforcement is significantly increased by the systemic (i.p.) administration of the pharmacological stressor yohimbine (0.0, 0.625, 1.25, 2.5 mg/kg) at the dose of 0.625 and 1.25 mg/kg. Values presented are the mean number of rewards earned in 30 min (±SEM). **(B)** Operant alcohol self-administration in Wistar (*n* = 10), as well as GG (*n* = 10), and AA (*n* = 10) msP rats under a progressive ratio (PR) schedule of reinforcement is significantly increased by the systemic (i.p.) administration of the pharmacological stressor yohimbine at the dose of 0.625 mg/kg. Values presented are the mean of break point measure (last ratio completed by the animal ±SEM). **p < 0.01, ***p < 0.001, significant difference from the collapsed means of vehicle-treated groups (0.0 mg/kg). Wistar: white bars; GG: gray bars; AA: black bars. For detailed statistics, see “Results.”

Although elevated level of alcohol consumption in msP rats is well known, overall ANOVA failed to show a main effect of “line” [*F*(2,31) = 0.8, NS], indicating that under the described experimental conditions alcohol-reinforced lever pressing was fairly equal between groups. A clear main effect of “treatment” [*F*(3,93) = 18.2, *p* < 0.001] that was not accompanied by a significant interaction “treatment × line” [*F*(6,93) = 1.5, NS] was also revealed to suggest that exposure to pharmacological stress similarly increased alcohol self-administration in all rat lines. On *post hoc* analysis of the collapsed variable of “treatment,” yohimbine significantly increased the number of alcohol rewards at doses of 0.625 (*p* < 0.001) and 1.25 mg/kg [(*p* < 0.01), Figure 2A].

### Yohimbine similarly increases break point of Wistar, as well as GG, and AA msP rats under a progressive schedule of reinforcement

To further explore how stress exposure interacts with the genetic background of the two msP lines, yohimbine at the dose of 0.625 mg/kg was tested on motivation to earn alcohol rewards as assessed by the PR schedule of reinforcement paradigm. Overall ANOVA showed a significant main effect of “line” [*F*(2,27) = 4.7, *p* < 0.05] accompanied by a significant main effect of treatment [*F*(1,27) = 23.7, *p* < 0.001] while interaction “treatment × line” was not significant [*F*(2,27) = 0.18, NS]. As revealed by *post hoc* analysis of the collapsed variable of “treatment,” these results suggest that 0.625 mg/kg of yohimbine clearly increased the break point measure in all three rat lines examined [(*p* < 0.001), Figure 2B].

### The AA line is more sensitive than other rat lines to the effect of antalarmin in reducing alcohol self-administration

**Figure 3:**
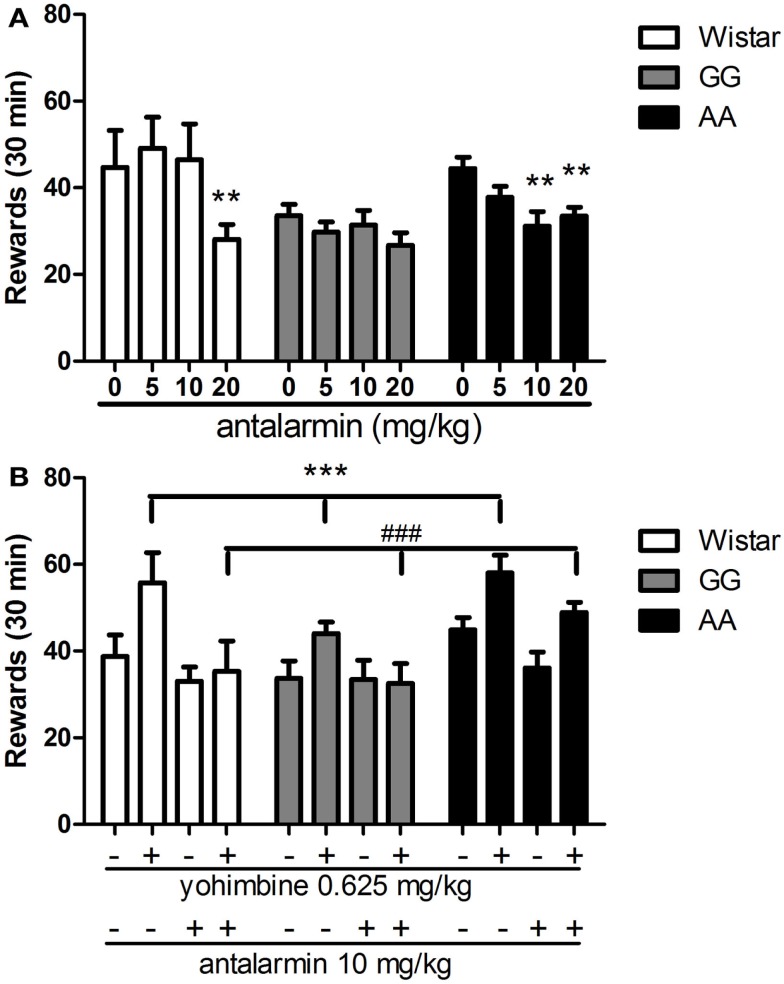
**(A)** Dose-response curve of antalarmin (0, 5, 10, 20 mg/kg) when systemically (i.p.) injected in Wistar (*n* = 7), as well as GG (*n* = 12), and AA (*n* = 14) msP rats as assessed on operant alcohol self-administration on a fixed ratio 3 (FR-3) schedule of reinforcement. The AA msP rat line shows increased sensitivity to antalarmin treatment compared to the other rat line examined. Data are the mean (±SEM) number of rewards earned in 30 min. **p < 0.01, difference from the vehicle-treated groups (0 mg/kg). **(B)** I.p. pre-treatment with antalarmin (10 mg/kg) fully blocks the escalation of alcohol self-administration (FR-3) elicited by systemic (i.p.) treatment with yohimbine at the dose of 0.625 mg/kg in all rat lines examined [Wistar (*n* = 8), as well as GG (*n* = 10) and AA (*n* = 15) msP rats]. Results are the mean (±SEM) number of rewards earned in 30 min. ***p < 0.001, difference from the groups receiving both vehicle-treatments of antalarmin and yohimbine (−/−); ^###^*p* < 0.001, difference from the groups receiving yohimbine 0.625 mg/kg (±). Wistar: white bars; GG: gray bars; AA: black bars. For detailed statistics, see “Results.”

As shown in Figure 3A, treatment with the CRF1-R antagonist antalarmin differentially reduced alcohol-reinforced lever pressing under FR-3 schedule. Overall ANOVA revealed a significant main effect of treatment [*F*(3,90) = 7.1, *p* < 0.001], significant main effect of “line” [*F*(2,30) = 4.5, *p* < 0.05] and significant interaction “treatment × line” [*F*(6,90) = 2.5, *p* < 0.05]. *Post hoc* analysis showed that antalarmin dose-dependently decreased lever pressing for alcohol in AA rats (*p* < 0.01 for doses of 10 and 20 mg/kg) while being ineffective in the GG line. Dose of 20 mg/kg antalarmin reduced the number of rewards in Wistar rats (*p* < 0.01).

### Yohimbine increases alcohol self-administration through a CRF-mediated mechanism

As shown in Figure 3B, pre-treatment with antalarmin blocked the yohimbine-induced increase of alcohol self-administration in all rat lines examined. Overall ANOVA showed a main effect of “treatment” [*F*(3,90) = 16.6, *p* < 0.001] accompanied by a main effect of “line” [*F*(2,30) = 3.8, *p* < 0.05] with no interaction “treatment × line” [*F*(6,90) = 1.4, NS]. In agreement with the experiments described above, *post hoc* analysis clearly revealed that yohimbine (0.625 mg/kg) significantly increased the number of alcohol rewards as compared to the collapsed means of the control groups (*p* < 0.001), and administration of antalarmin (10 mg/kg) fully prevented the effect of yohimbine (*p* < 0.001).

### Yohimbine at high dosages fails to produce reinstatement of alcohol seeking in AA msP rats

**Figure 4:**
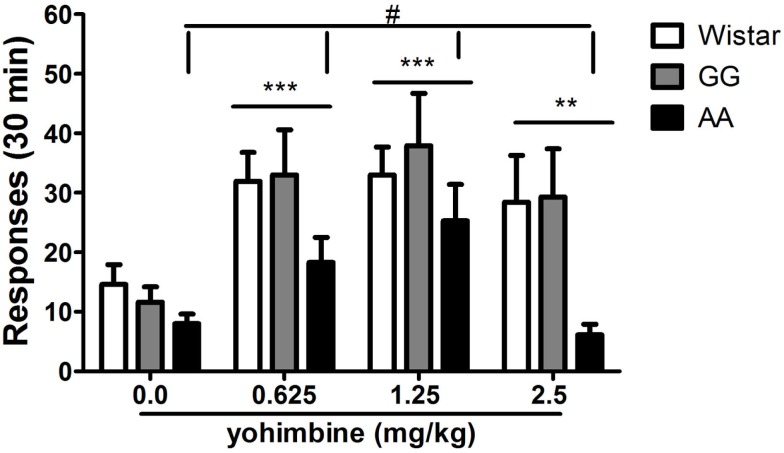
**Systemic (i.p.) administration of yohimbine (0.0, 0.625, 1.25, 2.5 mg/kg) elicits reinstatement of alcohol seeking in Wistar (*n* = 7), as well as GG (*n* = 8), and AA (*n* = 9) msP rats following extinction**. The AA msP line shows decreased threshold for yohimbine-induced reinstatement due to different sensitivity on responding to the effects of 2.5 mg/kg yohimbine dose. Data are the mean (±SEM) of total number of responses in 30 min. **p < 0.01, ***p < 0.001, difference from the vehicle-treated groups (0.0 mg/kg); ^#^*p* ≤ 0.05, difference from the collapsed means of both the GG msP and the Wistar lines. Wistar: white bars; GG: gray bars; AA: black bars. For detailed statistics, see “Results.”

The administration of yohimbine (0.0, 0.625, 1.25, 2.5 mg/kg) robustly reinstated responding on the previously alcohol-associated lever as shown by the significant main effect of “treatment” [*F*(3,63) = 11.6, *p* < 0.001]. Overall ANOVA also revealed a barely significant main effect of “line” [*F*(2,21) = 3.4, *p* = 0.05] and lack of the interaction “treatment × line” [*F*(6,63) = 0.8, NS]. These results suggest that all three rat lines examined were sensitive to the challenge of the pharmacological stressor. This was confirmed by *post hoc* analysis on the collapsed variable of “treatment” (0.625 and 1.25 mg/kg, *p* < 0.001; 2.5 mg/kg, *p* < 0.01). However, *post hoc* analysis conducted on the collapsed variable of “line” revealed that relapse-like behavior of the AA line was different from that of both the GG msP (*p* < 0.05) and the Wistar line (*p* = 0.05) following yohimbine treatment. This effect was the result of the fact that the AA msP line failed to reinstate the operant response following administration of 2.5 mg/kg. In contrast, both Wistars and GG msPs showed similar vulnerability to the pharmacological stressor as observed with lower dosages (Figure 4).

## Discussion

We found that the two msP rat lines (GG and AA) showed similar patterns of alcohol intake and preference in the 24-h access two-bottle free choice drinking paradigm, which was comparable to the elevated levels of drinking previously shown by the original msP line (Ciccocioppo et al., 2006; Hansson et al., 2007; Stopponi et al., 2011). In addition, stress exposure elicited increased operant alcohol self-administration in FR-3 and PR reinforcement schedules in both lines through a CRF1-R mediated mechanism. However, the msP line carrying the point mutations at the CRF1-R promoter region (AA) showed higher sensitivity than the wild type line (GG) to the effects of the CRF1-R blockade by the selective CRF1-R antagonist antalarmin. Also, the AA line showed altered vulnerability to relapse-like behavior following pharmacological stress exposure when compared to the GG line or to an unselected strain such as Wistar rats.

The observation that the two derived lines showed minimal changes in voluntary alcohol intake and preference suggests that the occurrence of the SNPs in the CRF1-R promoter region is not a causal genetic factor behind high alcohol intake. In operant situations, where rats work for alcohol reinforcement under limited-access conditions, results paralleled those obtained under unlimited 24-h voluntary alcohol access. However, in the present study, voluntary alcohol consumption was different between sub-lines only in 2 out of 15 days (days 6 and 13) where higher intake was observed in the AA line. This transient increase in the amount of drinking was associated with weekly cleaning of the animal room or exchange of sawdust. Thus, either increased arousal or heightened anxiety behavior may account for these isolated over drinking episodes. Indeed, msP rats are known to couple elevated alcohol consumption with comorbid anxiety which is thought to drive excessive drinking due to self-medication and tension relief purposes (Ciccocioppo et al., 2006; Ciccocioppo, 2013).

To test the hypothesis that stress exposure may contribute to confer functional relevance to the polymorphism, both AA and GG lines were exposed to pharmacological stress before self-administering alcohol as previously shown (Le et al., 2005; Marinelli et al., 2007). Induction of stress consisted of the administration of yohimbine, an alpha-2 adrenoceptor antagonist that increases noradrenaline cell firing (Aghajanian and VanderMaelen, 1982) and enhances noradrenaline release in terminal areas (Abercrombie et al., 1988; Pacak et al., 1992). Yohimbine induces anxiety-like responses in both humans (Holmberg and Gershon, 1961; Bremner et al., 1996b) and laboratory animals (Bremner et al., 1996a), and induced craving in alcohol-dependent patients (Umhau et al., 2011). Results of the present study demonstrate that yohimbine similarly increased alcohol-reinforced lever pressing in both rat lines, indicating that the polymorphism does not seem to play a major role in stress-induced alcohol drinking. These data were completed by the evidence that unselected Wistar rats showed a similar outcome as the derived msP lines when challenged with yohimbine under identical experimental conditions, a finding that closely paralleled results shown in previous studies (Le et al., 2005; Marinelli et al., 2007). In addition, the dose of yohimbine that increased alcohol self-administration under FR-3 schedule (0.625 mg/kg) in all three rat lines also increased the break point measure in all lines examined under the PR schedule, a paradigm known to better assess motivation to obtain a drug (Arnold and Roberts, 1997). This observation suggests that spontaneous occurrence of the polymorphism in msP animals does not appear to be associated with the exacerbated motivation to obtain alcohol following stress exposure.

The effect of yohimbine on increasing alcohol consumption shares some similarities with the effect of cycles of alcohol intoxication and withdrawal on inducing escalation of drinking (Rimondini et al., 2002; O’Dell et al., 2004; Gehlert et al., 2007; Walker and Koob, 2008; Gilpin and Koob, 2010), such that it has been hypothesized that yohimbine- and dependence-induced increases of operant alcohol self-administration may be mediated by similar neurobiological mechanisms (Marinelli et al., 2007). Firstly, both of these manipulations produce anxiety- and stress-like states (Breese et al., 2005a; Heilig and Koob, 2007). Secondly, both yohimbine treatment and alcohol dependence activate CRF system in structures of the extended amygdala (Merlo Pich et al., 1995; Zorrilla et al., 2001; Olive et al., 2002; Funk et al., 2006b; Sommer et al., 2008), brain areas thought to mediate the negative emotional state that leads to excessive alcohol use (Heilig and Koob, 2007; Koob, 2010; Breese et al., 2011). Lastly, antagonism at CRF1-R attenuates both yohimbine-induced (Marinelli et al., 2007) and dependence-induced increases of alcohol self-administration (Sabino et al., 2006; Chu et al., 2007; Funk et al., 2007; Gehlert et al., 2007). The observation that antalarmin prevented yohimbine-induced increase of alcohol operant responding in Wistar rats, as well as in the alcohol-preferring msP lines strongly supports a role of CRF-related mechanisms in the regulation of reinforcing effects of alcohol heightened by yohimbine treatment.

When antalarmin was tested under non-stressful conditions on the derived msP lines, the CRF1-R antagonist selectively reduced at doses of 10 and 20 mg/kg alcohol self-administration in the AA line, indicating that the polymorphism may confer sensitivity to this pharmacological manipulation. This observation parallels with what is previously shown in the original msP line where treatment with antalarmin reduced alcohol-reinforced lever pressing without altering that of unselected Wistar animals (Hansson et al., 2006). In that study, the differential effect of antalarmin on alcohol self-administration was associated with msP upregulation of CRF1-R expression and density, in turn linked to the occurrence of the point mutations in the CRF1-R gene. Thus, although data on CRF1-R expression or density of the AA versus GG line are not provided in the present study, it may be hypothesized that the selective reduction of operant responding for alcohol following antalarmin treatment in the AA line is due to upregulated CRF1-R function in these animals compared to the GG line. In addition, both the unique msP genetic profile and evidence showing that msP rats are, among other alcohol-preferring lines, the only one sensitive to CRF1-R antagonists (Ciccocioppo et al., 2006; Sabino et al., 2006; Gilpin et al., 2008) strongly supports the role of the polymorphism in eliciting increased sensitivity to the treatment with CRF1-R antagonists. Binding data on brain CRF1-R protein expression in AA and GG rats are needed to corroborate this hypothesis. Post-dependent animals were also shown to respond to this pharmacological treatment at doses that had no effects in non-dependent rats (Sabino et al., 2006; Chu et al., 2007; Funk et al., 2007; Gehlert et al., 2007) to suggest that the alcohol-dependent state recruits the CRF system. However, the CRF1-R signaling may be also be engaged when non-dependent animals escalate their levels of drinking (Sparta et al., 2008; Lowery et al., 2010; Cippitelli et al., 2012). Therefore, the reduction of alcohol self-administration observed in Wistar rats receiving the high dose of 20 mg/kg antalarmin is not surprising and may be due to abnormally elevated baseline of lever pressing of the cohort of animals employed in the present experiment. Of note, differences in operant alcohol drinking usually observed between msP and Wistar rats (Hansson et al., 2006; Gehlert et al., 2007) are not well reflected here probably due to different experimental conditions such as the use of an FR-3 reinforcement schedule. Previous studies employed an FR-1 schedule which may better reflect the rate of consumption as it delivers reinforcement after each response (Arnold and Roberts, 1997).

We have previously described that msP and unselected Wistar rats showed differential responses when exposed to increasing foot-shock stress intensities during extinction. Specifically, reinstatement of Wistar rats increased progressively with shock intensity while msPs reinstated responding on the previously alcohol-associated lever after low/medium but not high shock intensities which resulted in freezing behavior (Hansson et al., 2006). In the present study, a similar experiment that used different doses of yohimbine (0.625, 1.25, 2.5 mg/kg) instead of shock delivery was conducted to assess whether the polymorphism played a role on relapse-like behavior. Results showed that while yohimbine elicited reinstatement throughout the range of doses examined in both the GG line and the Wistar strain, animals carrying the polymorphism did not do so following injection of 2.5 mg/kg. This was likely due to highly stressed state of these rats and suggests that spontaneously occurring mutation at the CRF1-R gene may mediate an increased vulnerability to stress and possibly, mal-adaptive responses to intense stress exposure. MsP rats have anxiety and depression-like traits which are congruent to clinical alcoholism. Studies have shown that very high CRF1-R activation results in a passive behavior in anxiety models (Zhao et al., 2007; Tovote et al., 2010). As speculation, this inference could be extrapolated to our results where the AA rats, due to over-activated CRF signaling, were unable to reinstate responding at the highest yohimbine dose that may be able to further engage CRF system. However, by these data it is not possible to determine whether the polymorphism specifically regulates aspects of stress-induced alcohol seeking since CRF system has been shown to play a role in the reinstatement of various drugs of abuse (Shaham et al., 1997; Erb et al., 1998; Zislis et al., 2007) and natural rewards (Ghitza et al., 2006).

Alcoholism is a multi-genic disorder in which genetic predisposition combined with environmental factors may contribute to vulnerability to abuse. Studies have shown an association between alcoholism and several gene polymorphisms. For example, polymorphisms in the serotonin 2A receptor gene, dopamine transporter, μ-opioid, or GABA A receptor genes have been associated with alcohol dependence (Oslin et al., 2003; Edenberg and Kranzler, 2005; Ramchandani et al., 2011; Bhaskar et al., 2012; Wrzosek et al., 2012). In addition, recent clinical investigation has indicated the CRF1-R locus to mediate genetic susceptibility for excessive drinking (Treutlein et al., 2006). Polymorphisms in the CRF binding protein have also been associated with alcoholism (Enoch et al., 2008) and severity of stress-induced alcohol craving (Ray, 2011). Overall, these results suggest that incremental advances in treatment outcomes will result from an improved understanding of the genetic heterogeneity among patients with alcohol addiction that may ultimately lead to development of personalized treatments (Heilig et al., 2011). The present study may add to the field by providing evidence that spontaneously occurring mutations at the CRF1-R locus of msP animals acquire functional relevance leading to the expression of a particular phenotype which differs from that of animals with a normal genetic background.

## Conclusion

Here we show that two previously identified point mutations at the CRF1-R gene locus do not seem to play a major role in the expression of the msP excessive drinking phenotype or stress-induced drinking. However, their occurrence appears to be associated to an increased sensitivity to the effects of the pharmacological blockade of CRF1-R and to the decreased threshold for stress-induced reinstatement of alcohol seeking behavior. Despite the fact that there is no evidence for a correspondence of the same polymorphisms in msP rats and human alcoholics, these findings may have important pharmacogenetic implications because they suggest that only a subpopulation of alcoholics, the one characterized by specific mutation at CRF1-R gene or possibly carrying over-expression of the CRF1-R system, may respond to CRF1-R antagonists. Nowadays, this consideration is particularly relevant since there are ongoing clinical trials in which the efficacy of CRF1-R antagonists on alcohol addiction are under exploration (Zorrilla et al., 2013). On one hand, results of the present study may provide important inputs to the analysis of the clinical data that will soon be available. On the other hand, as it has already been demonstrated for naltrexone, a drug approved for the treatment of alcohol addiction, our results suggest that pharmacogenetic considerations are critical for appropriate clinical use of the agents (Heilig et al., 2011).

## Conflict of Interest Statement

The authors declare that the research was conducted in the absence of any commercial or financial relationships that could be construed as a potential conflict of interest.
